# Puerto Rico Coral Reef Monitoring Program Water Quality Data from 2023–2025

**DOI:** 10.1038/s41597-025-06468-6

**Published:** 2025-12-23

**Authors:** Travis A. Courtney, Martha Ricaurte-Chica, Adiana D. Bayo Torres, Aliyah M. Chabrier-Alpi, Ana G. Medina Martinez, Carolina V. Melendez Declet, Carla L. Mejías-Rivera, Carlos F. Fontanez Diaz, Catherine Hernández Rodríguez, Claudia I. Lebron-Maldanado, Erica Diaz Jimenez, Ivaneiliz Irizarry Torres, Juanita Carballeira Martínez, María José Brito-Vera, Irais Luquis Ramos, Idalmis Santos Echevarría, Johann J. Collazo Reyes, Leira J. Centeno Mejias, Manuel A. Nieves-Ortíz, Manuel F. Olmeda Saldaña, Margaret Gordon, Victoria Mendez Falcon, Victoria R. Reyes Claudio, Angela A. Garcia Fernandez, Angel R. Melendez Aguilar, Wanda E. Garcia Hernandez, Annette Feliberty Ruiz, Juan J. Cruz-Motta

**Affiliations:** 1 Department of Marine Sciences, University of Puerto Rico, Mayagüez, Mayagüez Puerto Rico; 2 Department of Chemical Engineering, University of Puerto Rico, Mayagüez, Mayagüez Puerto Rico; 3 School of Atmospheric and Earth Sciences, Georgia Institute of Technology, Atlanta, Georgia; 4 Department of Geology, University of Puerto Rico, Mayagüez, Mayagüez Puerto Rico; 5 Department of Natural and Environmental Resources, San Juan, Puerto Rico

**Keywords:** Marine chemistry, Marine chemistry

## Abstract

Coral reefs are declining due to a combination of global and local anthropogenic stressors. While water quality is an important driver of coral reef condition, the lack of systematic water quality monitoring efforts has prevented a more thorough analysis of the role of water quality parameters in modulating coral reef declines. Here we present 8,032 measurements representing seawater temperature, salinity, dissolved oxygen, pH_NBS_, Secchi depth, CO_2_ chemistry (dissolved inorganic carbon and total alkalinity), biological oxygen demand (BOD), total suspended solids, settleable solids, turbidity, chlorophyll-a, ortho-phosphate, nitrite + nitrate (NOx), total Kjeldahl nitrogen (TKN), and *Enterococcus* spp. from 42 Puerto Rico coral reef sites from 2023 to 2025. These data provide a critical baseline for coral reef water quality that can be used to develop and assess water quality thresholds, explore spatiotemporal variability in seawater chemistry, ground truth remote sensing observations, and downscale earth system models to improve global monitoring efforts and projections for coral reefs under ongoing anthropogenic impacts.

## Background & Summary

Coral reefs provide a range of ecosystem goods and services to humans worldwide^1^. However, mean coral cover has declined from 34.8% to just 16.3% from 1969 to 2011 while macroalgal cover increased from 7% to 23.6% during that same period^2^. Consequently, there are increasing concerns that coral reefs across the Caribbean are flattening^3^, may be unable to keep up with sea level rise^4,5^, and are decreasing their provisioning of ecosystem services^1,6^. Disease outbreaks, tourism, overfishing, and warming have been implicated as the primary drivers of declining Caribbean coral reef condition^2^. While sedimentation, heavy metals, herbicides, turbidity, nutrients, sewage, runoff, and fluvial inputs can also affect coral reef organisms and communities^7–10^, sparse water quality data has prevented a more robust analysis of its impacts on Caribbean coral reefs^2^.

**Fig. 1:**
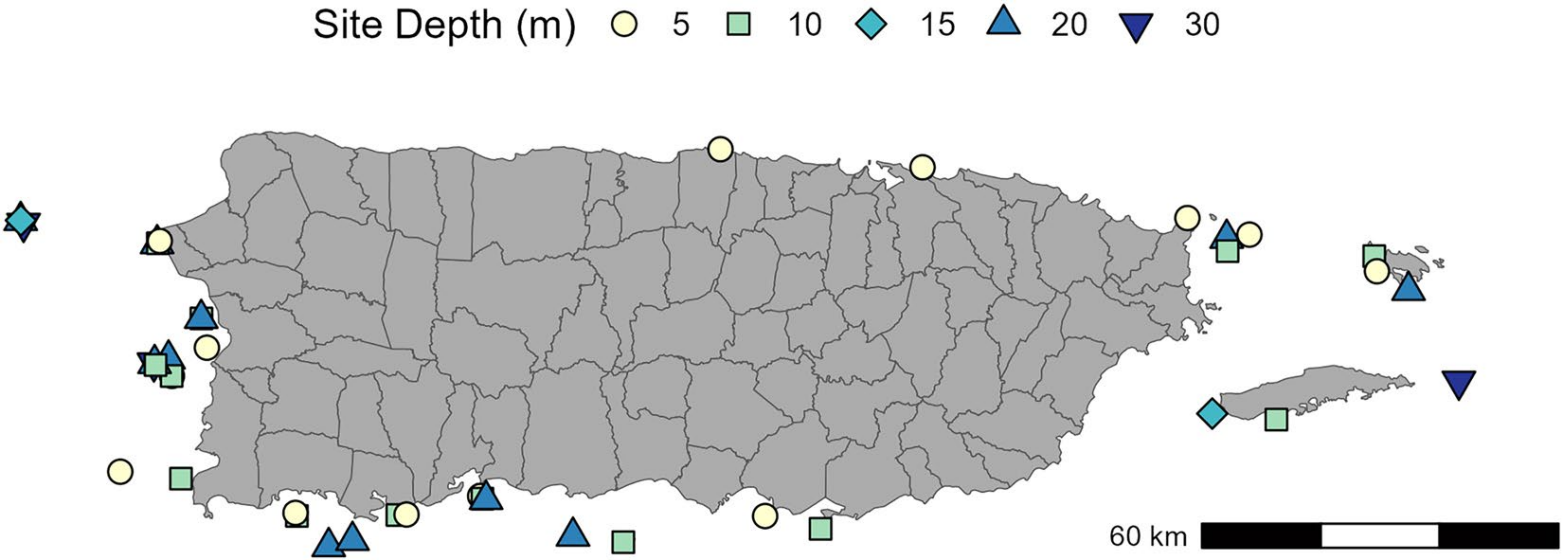
Map of the Puerto Rico Coral Reef Monitoring Program Sites (n = 42) surveyed in this data. Please note that several sites contain multiple sampling depths and appear as overlapping points.

In this study, we conducted eight quarterly water quality surveys from all of the Puerto Rico Coral Reef Monitoring Program (PRCRMP) sites from 2023–2025 for seawater temperature, salinity, dissolved oxygen, pH_NBS_, Secchi depth, CO_2_ chemistry (dissolved inorganic carbon and total alkalinity), biological oxygen demand (BOD), total suspended solids, settleable solids, turbidity, chlorophyll-a, ortho-phosphate, nitrite + nitrate (NOx), total Kjeldahl nitrogen (TKN), and *Enterococcus* spp. The PRCRMP was established in 1999 to monitor the benthic community composition and fish assemblages for selected coral reef sites ranging from 5 m to 30 m depth but have not been previously surveyed for water quality parameters (Fig. 1). To our knowledge, this data provides the first comprehensive snapshot of coral reef water quality across the Puerto Rico archipelago that can be used for a wide range of applications while serving as a baseline from which to evaluate future change.

## Methods

### Field Sampling

All field samples were collected via small boats navigating to the corresponding GPS coordinates for each station. At each site, seawater temperature, salinity, dissolved oxygen, and pH_NBS_ were recorded via handheld sonde (Ott Hydrolab HL4 prior to October 2023 or Eureka Manta + 20 following October 2023 with a YSI used as a backup for temperature and salinity in October 2023) at approximately 0.5 m depth for the surface and approximately 0.5 m above the seafloor for the bottom measurement. Temperature was measured following Standard Method 2550 B^11^, Salinity was measured following Standard Method 2520 B^11^, Dissolved Oxygen was measured following SM 4500-O H^11^, and pH was measured following EPA Method 150.2^12^. Calibration checks were performed daily and calibrated as needed to ensure a daily accuracy of depth (±0.4 m), temperature (±0.1°C), salinity (±0.5%), dissolved oxygen (±1.5%), and pH_NBS_ (±0.1) prior to each sampling event. A 20-cm Secchi disk was then deployed over the side of the boat three times, averaged, and reported to the nearest 0.5 m following US EPA LG-402^13^. When the bottom was clearly visible, the Secchi disk was not deployed and the Secchi depth was reported as being greater than the site depth.

An 8 L Van Dorn water sampler (General Oceanics Inc) was deployed to approximately 0.5 m depth to collect surface seawater for CO_2_ chemistry (dissolved inorganic carbon and total alkalinity) and biological oxygen demand (BOD). The same 8 L Van Dorn water sampler was then deployed to approximately 0.5 m above the bottom to collect bottom seawater for CO_2_ chemistry (dissolved inorganic carbon and total alkalinity), biological oxygen demand (BOD), total suspended solids, settleable solids, turbidity, chlorophyll-a, ortho-phosphate, nitrite + nitrate (NOx), total Kjeldahl nitrogen (TKN), and *Enterococcus* spp. All seawater samples were sampled into their respective bottles, placed in coolers upon collection, and kept cool during transport to the Caribbean Coral Reef Institute Water Quality Laboratory at the Department of Marine Sciences, University of Puerto Rico Mayagüez for subsequent analyses. All samples were collected and analyzed according to standard methods for the analysis of coastal waters (Table 1) with laboratory assessment for precision and accuracy. Additionally, 10% of all field samples were collected in duplicate to assess field-based precision.

**Table 1:** Laboratory mean percent error relative to known standards and mean relative percent difference for field duplicates are reported for each parameter.

Parameter	Units	Mean Percent Error	Mean Relative Percent Difference
Temperature	°C	0.02	−0.06
Salinity	psu	−0.29	−0.05
DO	mg/L	0.22	0.57
pH	N/A	−0.26	0.02
Secchi Depth	m	n.d.	1.75
Dissolved Inorganic Carbon	µmol/kg	−0.44	−0.04
Total Alkalinity	µmol/kg	−0.18	−0.04
Biological Oxygen Demand	mg/L	−26.5	6.29
Settleable Solids	mL/L	n.d.	0
Total Suspended Solids	mg/L	−2.13	−1.54
Turbidity	NTU	n.d.	3.97
Chlorophyll-a	µg/L	4.35	6.34
ortho-Phosphate	mg/L	−2.07	3.82
Nitrate + Nitrite	mg/L	−4.99	5.22
Total Kjeldahl Nitrogen	mg/L	−3.7	5.42
*Enterococcus* spp.	ind/100 mL	n.d.	32.23

No data (n.d.) exists for the mean percent error calculations for Secchi Depth, *Enterococcus* spp., and Settleable Solids owing to the absence of certified reference materials. While Turbidity was evaluated to within 10% of certified reference values daily, only one daily calibration check with a mean accuracy of 0.8% was recorded in Quarter 1 so the mean percent error was reported as n.d. for this parameter owing to the lack of consistent checks across all samples.

### Lab Analyses

Seawater CO_2_ chemistry was analyzed following best practices^14^. Each sample was collected in 150 mL screw-top Pyrex glass bottles, immediately fixed with 50 μL mercury (II) chloride (HgCl_2_), and kept dark and cool until analysis for dissolved inorganic carbon (DIC) on an Apollo Scitech AS-C5 Dissolved Inorganic Carbon Analyzer and total alkalinity (TA) on an Apollo Scitech AS-ALK3 Alkalinity Unit. All analyses were conducted in units of µM and converted to µmol/kg using calculated seawater density^15^ using the temperature measured at the time of analysis by a NIST-certified thermistor and seawater salinity measured *in situ* by the handheld sonde. Both DIC and TA were analyzed relative to certified reference materials provided by the laboratory of Prof. Andrew Dickson to assess accuracy and precision. CO_2_ chemistry samples were typically analyzed within 28 days of collection.

Biological oxygen demand (BOD) was analyzed following Standard Method 5210 B^11^. Each sample was collected in 1 L plastic bottles and was well-aerated before being placed in 300 mL borosilicate BOD bottles with 300 μL each of prepared phosphate buffer, MgSO_4_, CaCl_2_, and FeCl_3_ solutions at 20 °C ± 1 °C for 5 days (±6 hours). Dissolved oxygen was measured before and after the 5-day incubation period using a Thermo Scientific Orion Auto Star A213 benchtop RDO/DO meter with Thermo Scientific Orion AUTO-STIR BOD Probe calibrated daily to water saturated air. BOD was then calculated by the following equation:

$${Biological\; Oxygen\; Demand}({mg}/L)={{DO}}_{{initial}}({mg}/L)-{{DO}}_{{final}}({mg}/L)$$

where DO_initial_ (mg/L) was the dissolved oxygen measured prior to the incubation and DO_final_ (mg/L) was the dissolved oxygen concentration measured after the incubation. Due to a malfunction of the AUTO-STIR BOD probe, the DO for several samples was measured by the Ott Hydrolab HL4 and a non-stirring DO probe in Quarter 5. Laboratory precision was assessed using laboratory replicates and laboratory accuracy was assessed via the addition of a 2 mg/L BOD spike from a Glucose Glutamic Acid solution added to a laboratory replicate (except for the first quarterly sampling).

Settleable solids were analyzed following Standard Method SM 2540-F^11^. Each sample was collected into a 1 L polypropylene plastic bottle, kept cool, and analyzed within 48 hours of sample collection. Prior to analysis, samples were brought to room temperature and shaken. The sample was then poured into an Imhoff cone and allowed to settle for 45 minutes. It was then gently agitated, and left to settle for an additional 15 minutes. The volume of settled solids at the bottom of the cone was recorded in units of mL/L.

Total suspended solids were analyzed following Standard Method 2540-D^11^. Each sample was collected into a 1 L polypropylene plastic bottle, kept cool, and analyzed within 48 hours of sample collection. Prior to analysis, the sample was gently mixed and poured into a graduated cylinder to record the actual sample volume. The sample was then completely filtered through a 47 mm Whatman pre-weighed, ready-to-use, glass microfiber filter (1.5 μm particle retention), dried at 104°C for at least 1 hour, cooled in a desiccator for at least 15 minutes, and then weighed on a Mettler Toledo ME204T Balance (±0.1 mg). This drying, cooling, and weighing cycle was repeated until a constant weight was achieved (i.e., difference of less than 0.5 mg between replicate weights). Total suspended solids were then calculated by the following equation:

$${Total\; Suspended\; Solids}\left({mg}/L\right)=\frac{\left(A-B\right)\times 1000}{{sample\; volume}({ml})}$$

where A is the final weight (mg), B is the initial weight (mg), sample volume is the filtered sample volume (mL), and 1000 converts to units of mg/L. Laboratory accuracy was assessed at least every 20 samples by processing 1 L of reagent water (0 mg/L) and 1 L of reagent water with 100 mg cellulose (100 mg/L) following the same procedures.

Turbidity was analyzed following EPA Method 180.1^12^. Each sample was collected into a 250 mL polypropylene plastic bottle, kept cool, and analyzed within 48 hours of sample collection. All samples were brought to room temperature and analyzed on an Orion AQ5400 Portable Turbidimeter. Results were reported to the nearest 0.05 for samples between 0 and 1.0 NTU and the nearest 0.1 for samples ranging from 1 and 10 NTU. Calibration checks were performed daily prior to sample analysis using Orion formazin standard solutions (0, 1, 10, 100, 1000 NTU) to ensure accuracy (blank < 0.1 NTU and standards within 10%), but, regrettably, only the first calibration check was recorded.

Chlorophyll-a was analyzed following Standard Method 10200 H^11^. Each sample was collected into a 1 L amber glass bottle and kept cool and dark until sample processing within 24 hours of collection. Immediately prior to sample processing, 2 mL of saturated MgCO_3_ solution was added to each sample. The sample was then filtered through a 47 mm Whatman GF/F Glass Microfiber Filter (0.7 μm particle retention), and the filtrate was measured using a graduated cylinder for subsequent calculation of concentrations. The filter was preserved in aluminum foil at −20 °C until analysis within 28 days. While most samples were filtered at the Caribbean Coral Reef Institute Water Quality Laboratory, several chlorophyll-a samples were filtered in a mobile laboratory using the same equipment to ensure analysis began within 24 hours of sample collection. Each filter was ground in a 90% acetone solution, steeped for 2 hours, centrifuged for 20 minutes at 500 G, and the resulting supernatant was analyzed in a 1 cm cuvette on an Orion AquaMate 8100 UV-Vis spectrophotometer at 664.0, 665.0, and 750.0 nm before and after the addition of 100 μl of 0.1 N HCl. Chlorophyll-a was then calculated via the following equation:

$${Chlorophyll}\mbox{-}a({\rm{\mu }}g/L)=\frac{26.7[\left({664}_{b}-{750}_{b}\right)-\left({665}_{a}-{750}_{a}\right)]\times {V}_{1}}{{V}_{2}\times L}\times 1000$$

where 26.7 is the absorbance correction, X_b_ represents the absorbance for the respective wavelength before the HCl addition, X_a_ represents the absorbance for the respective wavelength after the HCl addition, V_1_ is the volume of the sample extract (mL), V_2_ is the volume of the sample (mL), L is the width of the cuvette (1 cm), and 1000 converts from units of mg/L to µg/L. Laboratory accuracy was assessed via certified reference material (Turner Designs, Pyxis) on each day of analysis.

Dissolved inorganic nutrients were analyzed simultaneously for ortho-phosphate following EPA Method 365.1^12^ and for nitrite + nitrate (NOx) following EPA Method 353.2^12^. Samples were collected in 125 ml polypropylene plastic bottles and kept cool during transport to the lab. They were then acidified to a pH of 2 using sulfuric acid and preserved at ~−4 °C until analysis within ~28 days of sample collection. Prior to analysis, samples were neutralized to a pH of 7 using a sodium hydroxide solution and filtered through a 28 mm Sartorius minisart High Flow PES 0.45 μM membrane filter. Samples were analyzed spectrophotometrically on a SEAL AA500 AutoAnalyzer relative to freshly mixed laboratory standards. Laboratory accuracy was assessed relative to certified reference material (Sigma Aldrich for Quarter 1, ERA Waters thereafter), spiked samples, and spiked blanks analyzed every 10 samples during each analytical run.

Total Kjeldahl nitrogen (TKN) was analyzed following EPA Method 351.2^12^. Samples were collected in 50 mL centrifuge tubes and kept cool during transport to the lab. They were then acidified to a pH of 2 using sulfuric acid and preserved at ~−4 °C until analysis within ~28 days. Samples, calibrants, blanks, CRM, and spikes were digested using a SEAL BD50 Block Digestor in a sulfuric acid digestion solution containing H_2_SO_4_, K_2_SO_4_, and CuSO_4_ at 380°C for 1.5 hours. The resulting samples were allowed to cool to room temperature and were then diluted with 25 mL of distilled water for analysis. Samples were filtered through a 28 mm Sartorius minisart High Flow PES 0.45 μM membrane filter prior to spectrophotometric analysis on a SEAL AA500 AutoAnalyzer relative to the digested standards. Laboratory accuracy was assessed relative to certified reference material (Sigma Aldrich), spiked samples, and spiked blanks analyzed every 10 samples during each analytical run. Quarter 2 samples were analyzed using an incorrect standard calibration curve and were subsequently corrected by applying a post-analysis correction factor of 0.21 to correct for this error.

*Enterococcus* spp. was analyzed following Standard Method 9230-D^11^. Each sample was collected into a sterile Whirl-Pak bag, kept cool, and analyzed within 6 hours of sample collection. While most samples were analyzed at the Caribbean Coral Reef Institute Water Quality Laboratory, several *Enterococcus* spp. samples were analyzed in a mobile laboratory to ensure analysis began within 6 hours of sample collection. A 10 mL aliquot of each sample was added to 90 mL sterilized ultrapure water in an IDEXX vessel and mixed with IDEXX Enterolert reagent. Each sample was then mixed, poured into a IDEXX Sterile Quanti-Tray/2000, sealed, and incubated at 41 ± 0.5 °C for 24 to 28 hours. Sample wells were then illuminated by UV-light to count the number of large and small fluorescent wells. The most probable number was determined by the number of fluorescent wells using the IDEXX Enterolert tables and was multiplied by 10 to account for the 1/10 dilution to report the results in units of most probable number per 100 mL (MPN/100 mL). Each batch of IDEXX Enterolert test media was internally validated using a blank (no inoculation), positive (*Enterococcus faecium*), and negative (*Serratia marcescens, Aerococcus viridans*) inoculation.

### Data processing

The field and laboratory databases were merged for each sampling cycle using *R* and then manually appended and reorganized in *Excel* to create the complete database. The final database consists of a single comma-separated values file where columns indicate variables and rows indicate observations for each site surveyed. We used *NA* for any missing observations either due to an absence of sample collection, inadequate sample preservation, or analyses that failed quality method-specific assurance criteria and were removed from the database. For example, there were several instances of either erroneous measurements, unstable readings, or failure to collect any data by the Ott Hydrolab HL4 sonde despite passing daily calibration checks. These unstable measurements resulted in erroneously high or low field measurements that have been omitted from the final data, resulting in data gaps for Temperature, Salinity, Dissolved Oxygen, and pH primarily in Quarters 1 and 2. Total Alkalinity is largely missing for Quarters 1 and 2 due to analytical precision that exceeded the desired 4 µmol/kg accuracy, with insufficient samples to re-analyze. Several Dissolved Inorganic Carbon samples were opened but could not be analyzed that day owing to instrument failures and consequently were compromised by gas exchange in the lab. Many of the dissolved inorganic nutrients samples were also less than the blank seawater in Quarters 1 and 2 and were consequently reported as less than the blank measurement. Subsequent Quarters were analyzed with purer source water and combined with the analysis of 10 repeated blanks to formally quantify detection limits following EPA Methods^12^. Additionally, there were two elevated Chl-a samples and one elevated ortho-phosphate sample that were abnormally high and suggested potential sample contamination. Because all other quality control measures were met and the samples were not available for re-analysis for these Chl-a and ortho-phosphate measurements, we substituted these samples with a “R” and preserved the seemingly erroneous corresponding value in the final “Removed” data column. We used “*< X*” for any samples that were less than our limit of detection (*X*) and, similarly, “*> X*” for any Secchi depths that exceeded site depth (*X*). The raw data sheets and lab reports for the analysis of all parameters, including the erroneous measurements not included in the final database, are provided separately for each sampling Quarter in the associated code release.

## Data Records

The dataset is publicly available via *FigShare*^16^ and consists of a single comma-separated values file. The complete list of columns and their corresponding metadata are listed below:

*Sampling_Cycle* refers to the number of each quarterly sampling event

*Site_Name* refers to the name and corresponding depth of each of the PRCRMP sites

*Location* refers to the nearest municipality in Puerto Rico where the site is located

*Site_Code* refers to the PRCRMP site code

*Region *refers to the location of the site within the main island of Puerto Rico or its sub-islands

*Zone* refers to the cardinal direction of the sampling site with respect to Puerto Rico

*Latitude* refers to the latitude of the sampling site

*Longitude* refers to the longitude of the sampling site

*Depth* refers to the depth of the sampling site

*Month* refers to the month of sample collection

*Day* refers to the day of sample collection

*Year* refers to the year of sample collection

*Date* refers to the date of sample collection in MM/DD/YYYY format

*Time_In* refers to the starting local time (AST) of sample collection in 24-hour format HH:MM

*Time_Out* refers to the ending local time (AST) of sample collection in 24-hour format HH:MM

*Surface_Temp_C* refers to the surface seawater temperature in °C

*Surface_Temp_Depth_m* refers to the depth in meters of the surface temperature measurement

*Surface_Sal_psu* refers to the surface seawater salinity in units of psu

*Surface_Sal_Depth_m* refers to the depth in meters of the surface salinity measurement

*Surface_DO_mg_L* refers to the surface dissolved oxygen in units of mg/L

*Surface_DO_Depth_m* refers to the depth in meters of the surface dissolved oxygen measurement

*Surface_pH* refers to the surface seawater pH on the NBS scale

*Surface_pH_Depth_m* refers to the depth in meters of the surface pH measurement

*Bottom_Temp_C* refers to the bottom seawater temperature in °C

*Bottom_Temp_Depth_m* refers to the depth in meters of the bottom temperature measurement

*Bottom_Sal_psu* refers to the bottom seawater salinity in units of psu

*Bottom_Sal_Depth_m* refers to the depth in meters of the bottom salinity measurement

*Bottom_DO_mg_L* refers to the bottom dissolved oxygen in units of mg/L

*Bottom_DO_Depth_m* refers to the depth in meters of the bottom dissolved oxygen measurement

*Bottom_pH* refers to the bottom seawater pH on the NBS scale

*Bottom_pH_Depth_m* refers to the depth in meters of the bottom pH measurement

*Secchi_Depth_m* refers to the mean measured Secchi depth in meters

*Surface_Van_Dorn_Depth_m* refers to the collection depth of surface chemistry samples

*Bottom_Van_Dorn_Depth_m* refers to the collection depth of the bottom chemistry samples

*Surface_DIC_umol_kg* refers to the surface-collected dissolved inorganic carbon in µmol/kg

*Bottom_DIC_umol_kg* refers to the bottom-collected dissolved inorganic carbon in µmol/kg

*Surface_TA_umol_kg* refers to the surface-collected total alkalinity in µmol/kg

*Bottom_TA_umol_kg* refers to the bottom-collected total alkalinity in µmol/kg

*Surface_BOD_mg_L* refers to the surface-collected biological oxygen demand in mg/L

*Bottom_BOD_mg_L* refers to the bottom-collected biological oxygen demand in mg/L

*Bottom_SS_ml_L* refers to the bottom-collected settleable solids in mL/L

*Bottom_TSS_mg_L* refers to the bottom-collected total suspended solids in mg/L

*Bottom_Turbidity_NTU* refers to the bottom-collected turbidity in NTU

*Bottom_Chl-a_ug_L* refers to the bottom-collected chlorophyll-a in µg/L

*Bottom_PO4_mg_L* refers to the bottom-collected ortho-phosphate in mg/L

*Bottom_PO4_LOD_mg_L* refers to the bottom-collected ortho-phosphate in mg/L relative to the limit of detection for reporting

*Bottom_NOX_mg_L* refers to the bottom-collected nitrate + nitrite in mg/L

*Bottom_NOx_LOD_mg_L* refers to the bottom-collected nitrate + nitrite in mg/L relative to the limit of detection for reporting

*Bottom_TKN_mg_L* refers to the bottom-collected total Kjeldahl nitrogen in mg/L

*Bottom_TKN_LOD_mg_L* refers to the bottom-collected total Kjeldahl nitrogen in mg/L relative to the limit of detection for reporting

*Bottom_Enterococcus_MPN_100mL* refers to the bottom-collected most probable number of *Enterococcus* spp. per 100 mL

*Bottom_Enterococcus_LOD_ MPN_100mL* refers to the bottom-collected most probable number of *Enterococcus* spp. per 100 mL of sample relative to the limit of detection for reporting

*Removed* refers to the corresponding value denoted by *R* that was removed from that row

## Technical Validation

Laboratory accuracy was calculated for each parameter as the percent error of measured values relative to the standard value reported for the certified reference materials using the following equation:

$${Percent\; Error}( \% )=\frac{{Measured}-{Standard}}{{Standard}}\times 100$$

where *Measured* is the value measured in the lab and *Standard* is the value reported for the reference material. The mean percent error was determined from the percent error across all Quarters for each parameter (Table 1).

Field precision was calculated for each parameter as the relative percentage difference (RPD) between field measurements as per the following equation:

$${Relative\; Percent\; Difference}( \% )=\frac{{Sample\; Value}-{Replicate\; Value}}{\left(\frac{{Sample\; Value}+{Replicate\; Value}}{2}\right)}\times 100$$

where the *Sample Value* refers to the primary collected data and *Replicate Value* refers to the replicate sample value for each parameter and 100 converts from a proportion to a percentage (Table 1). The mean relative percent difference was determined from the relative percent difference across all Quarters for each parameter (Table 1).

## Data Overview

Summary statistics for each of the measured parameters across the entire dataset were generated using the *R* function *describe* in the *psych*^17^ package (Table 2).

**Table 2:** Summary statistics are provided for each parameter across the full data set.

Parameter	Depth	n	Mean	SD	Min	Max
Temperature (°C)	S	371	28.94	1.26	26.34	31.25
	B	369	28.85	1.26	26.35	31.15
Salinity (psu)	S	363	36.31	0.90	33.37	38.07
	B	361	36.38	0.88	33.19	38.08
Dissolved Oxygen (mg/L)	S	371	6.24	0.35	4.37	8.24
	B	369	6.13	0.41	3.87	8.39
pH (NBS)	S	331	8.15	0.05	8.00	8.29
	B	342	8.15	0.05	8.00	8.27
Secchi Depth (m)	N/A	129	10.20	4.66	2.67	23.67
Dissolved Inorganic Carbon (µmol/kg)	S	349	2005.81	28.20	1911.01	2073.00
	B	353	2008.06	28.93	1927.15	2196.08
Total Alkalinity (µmol/kg)	S	299	2345.54	38.01	2162.00	2437.00
	B	297	2346.27	38.90	2162.00	2444.00
Biological Oxygen Demand (mg/L)	S	374	0.53	0.52	−0.08	3.78
	B	374	0.52	0.55	−0.12	4.41
Settleable Solids (mL/L)	B	374	0.00	0.02	0.00	0.30
Total Suspended Solids (mg/L)	B	374	7.31	6.04	0.00	60.00
Turbidity (NTU)	B	374	0.17	0.31	0.00	4.45
Chlorophyll-a (µg/L)	B	372	0.42	0.33	0.00	2.51
ortho-Phosphate (mg/L)	B	369	0.01	0.02	0.00	0.46
Nitrate + Nitrite (mg/L)	B	372	0.01	0.01	−0.01	0.13
Total Kjeldahl Nitrogen (mg/L)	B	371	0.09	0.26	−0.75	1.06
*Enterococcus* spp. (ind/100 mL)	B	374	7.7	43.76	0	697

Depth represents the depth of collection where S = Surface and B = Bottom, n represents the total number of measurements, mean represents the mean of all measurements, SD represents the standard deviation of all measurements, Min represents the minimum measured value, and Max represents the maximum measured value for each of the 16 parameters. Note that Secchi Depth *n* is significantly lower than expected because the Secchi depth exceeded the site depth for most sites and Total Alkalinity *n* is lower than Dissolve Inorganic Carbon *n* due to insufficient sample volume required to repeat analyses exceeding desired 4 µmol/kg accuracy during Quarters 1 and 2.

## Usage Notes

These data provide a baseline for water quality parameters for the coral reefs of Puerto Rico, building on similar assessments conducted elsewhere in the Caribbean^8,18,19^ to improve our understanding of patterns of temporal and spatial variation of water quality parameters on Caribbean coral reefs^2^. By assessing these data relative to coral reef bioindicators^7,20^ at the PRCRMP sites, these data could potentially also be used to develop local water quality guidelines (e.g.^21^) to help inform local management and conservation efforts in Puerto Rico and across the Caribbean. The spatial and temporal variability of water quality parameters is also intrinsically valuable for understanding coral reef metabolism and processes (e.g.^22–26^). In addition to their direct assessments of water quality, these data can also provide important ground truthing of remotely sensed parameters^27^ and optimization of earth system models^28^ to improve global monitoring efforts and projections for coral reefs under ongoing anthropogenic impacts.

## Data Availability

All raw data, reports, R code, and Excel sheets used to compile the primary comma-separated values data file described here and copies of the Quality Assurance Project Plan, laboratory-specific Standard Operating Procedures, calibration checks, and data sheets associated with each parameter are publicly available on *Zenodo*: 10.5281/zenodo.17582612.

## Data availability

The dataset is publicly available on *FigShare:* 10.6084/m9.figshare.30591008.v1.
